# The Significance of 18F-Fluorocholine-PET/CT as Localizing Imaging Technique in Patients with Primary Hyperparathyroidism and Negative Conventional Imaging

**DOI:** 10.3389/fendo.2017.00380

**Published:** 2018-01-22

**Authors:** Stefan Fischli, Isabelle Suter-Widmer, Ba Tung Nguyen, Werner Müller, Jürg Metzger, Klaus Strobel, Hannes Grünig, Christoph Henzen

**Affiliations:** ^1^Division of Endocrinology, Diabetes and Clinical Nutrition, Luzerner Kantonsspital, Luzern, Switzerland; ^2^Division of Otorhinolaryngology, Head and Neck Surgery, Luzerner Kantonsspital, Luzern, Switzerland; ^3^Division of Visceral Surgery, Luzerner Kantonsspital, Luzern, Switzerland; ^4^Division of Nuclear Medicine/Radiology, Luzerner Kantonsspital, Luzern, Switzerland

**Keywords:** primary hyperparathyroidism, 18F-fluorocholine-PET/CT, localization diagnostic, sestamibi scintigraphy, cervical ultrasound, parathyroidectomy, multinodular goiter

## Abstract

**Objective:**

The essential prerequisite for focused parathyroidectomy in patients with primary hyperparathyroidism (pHPT) is proper localization of all autonomic tissue. Sensitivity of conventional imaging modalities (ultrasound, ^99m^Tc-sestamibi scintigraphy/SPECT/CT) is influenced by different factors (i.e., size/weight and position of autonomic tissue) and decreases in the presence of a multinodular goiter. Therefore, a considerable percentage of pHPT patients have negative or equivocal localization studies before surgery. The aim of this study is to evaluate the utility of FCH-PET/CT for preoperative localization in patients with pHPT and negative/equivocal ^99m^Tc-sestamibi scintigraphy/SPECT/CT and/or ultrasound.

**Methods and measurements:**

Between 2014 and 2017, a total of 39 patients with pHPT and negative/equivocal conventional imaging were referred for FCH-PET/CT. In the analysis, we included those (*n* = 23) who had surgery and a histopathologic workup of the lesions.

**Results:**

19 of 23 patients demonstrated no tracer uptake with ^99m^Tc-sestamibi scintigraphy/SPECT/CT, 6 patients had an equivocal sonographic lesion, and multinodular goiter was present in 43% (10/23). In 21 of 23 patients, hyperfunctioning parathyroid tissue was identified correctly by FCH-PET/CT [21 true positive, 1 false negative, and 1 false positive; per-patient sensitivity 95.5% (95% confidence interval {CI}, 77.2–99.9)]. 29 lesions were resected [21 true positives, 3 false negatives, 1 false positive, and 4 true negatives; per-lesion sensitivity 87.5% (95% CI, 67.6–97.3)]. All patients were classified as having surgical success according to a decrease of intraoperative parathyroid hormone of ≥50% and normalization of postoperative serum calcium levels.

**Conclusion:**

Despite a high prevalence of multinodular goiter, diagnostic accuracy of FCH-PET/CT in our patient group was excellent. Therefore, FCH-PET/CT is a promising new imaging tool in patients with pHPT and negative/equivocal results by conventional imaging techniques.

## Introduction

Primary hyperparathyroidism (pHPT) is characterized by the autonomous secretion of parathyroid hormone (PTH) by one or more parathyroid glands. The most frequent cause of pHPT, occurring in 80–90% of patients, is a benign solitary parathyroid adenoma (1). However, multiple glandular disease occurs in 10–20% of patients, either as a double adenoma or as a multiple glandular hyperplasia (1, 2). The latter typically occurs in familiar forms of pHPT (e.g., MEN 1/2 or CDKN1B mutations) or in patients treated with lithium. An additional fifth parathyroid gland is found in 6–15% of patients (3, 4). Due to migration during embryological development, the anatomical localizations of parathyroid glands are highly variable. Ectopic localizations are found in ≤20% of patients in an autopsy series (5), with an even higher prevalence (≤50%) in patients with persistent or recurrent pHPT after surgery (6, 7).

Surgery is the only definitive cure for pHPT and is indicated in all symptomatic patients and in a defined subgroup of “asymptomatic” patients (8). The goals of parathyroid surgery are to remove all hyperfunctioning tissue and to preserve normal parathyroid glands to prevent postoperative hypoparathyroidism. In the past, the standard surgical approach included bilateral cervical exploration regardless of preoperative imaging studies. Minimally invasive or focused approaches have currently gained more acceptance as safe and effective alternatives (9–11), with the advantages of a shorter duration of surgery, a reduction of tissue damage (7), and a lower complication rate (12–15). The essential prerequisite for focused parathyroidectomy is proper localization of all autonomic tissue, including detection of ectopic and super numerous parathyroid lesions.

The most commonly used imaging modalities for this purpose are ultrasounds of the neck and parathyroid scintigraphy with ^99m^Tc-sestamibi (15, 16). Cervical ultrasound has a reported sensitivity ranging from 64 to 89% (17, 18); however, the detection rate is operator dependent and is lower in patients with multiple glandular disease and with multinodular goiters (2, 19). In addition, sonographic assessment of ectopic lesions is limited to retrotracheal or retroesophageal localizations, or even impossible in the case of an intrathoracic adenoma.

The technique of ^99m^Tc-sestamibi scintigraphy, often in combination with SPECT/CT, is considered the reference method for preoperative parathyroid imaging, with a sensitivity of 70–88% (2, 17, 18). As in ultrasound, the sensitivity is reduced in patients with multiglandular disease and multinodular goiter, and the diagnostic accuracy depends on the size and weight of the parathyroid gland (19, 20). However, even in the presence of a single uptaking focus in scintigraphy, multiglandular disease cannot be precluded (21). Even combined imaging with ultrasound and ^99m^Tc-sestamibi scintigraphy/SPECT/CT leaves a considerable percentage of pHPT patients with negative or equivocal localization studies before surgery, in which cases, successful parathyroidectomy depends solely on the expertise of the surgeon.

Recent patient level studies have shown good accuracy and equal or even superior detection using 18F-fluorocholine-PET/CT (FCH-PET/CT) compared to commonly used imaging using ^99m^Tc-sestamibi scintigraphy, ultrasound, and four-dimensional computed tomography (21–24). In this study, we therefore evaluated the sensitivity and specificity of FCH-PET/CT for preoperative localization in patients with pHPT and negative or equivocal ^99m^Tc-sestamibi scintigraphy and/or ultrasound.

## Subjects and Methods

**Figure 1:**
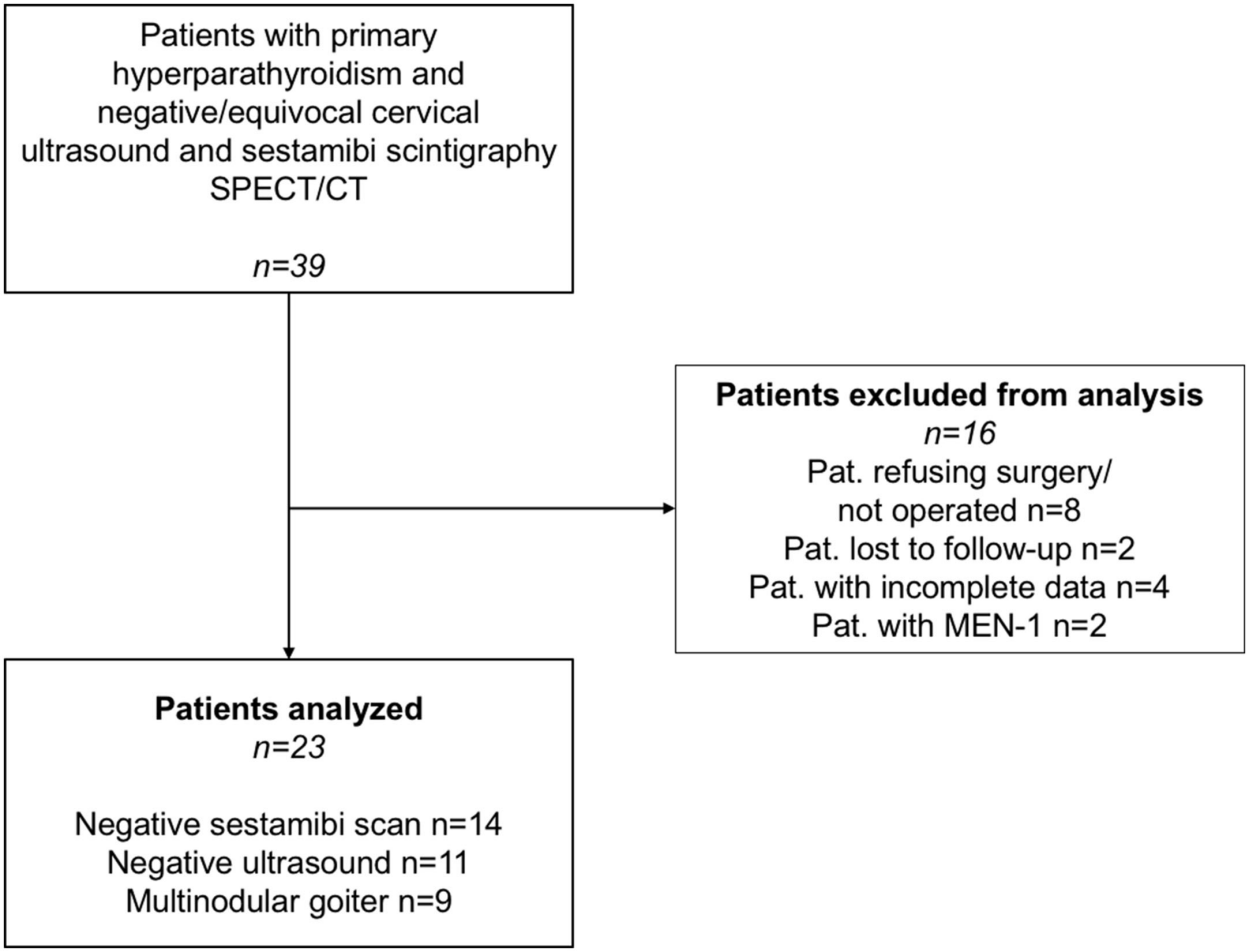
Screening and enrollment of patients.

This was a retrospective cohort study from January 2014 to March 2017 that included patients with pHPT older than 18 years who were candidates for focused parathyroidectomy, as defined by the current guidelines (8). All patients had negative or equivocal localization studies with ^99m^Tc-sestamibi scintigraphy/SPECT/CT and were offered additional imaging with FCH-PET/CT. Written consent was obtained from all participants after they were informed that FCH-PET/CT was an authorized diagnostic imaging procedure for indications other than parathyroid disease. PTH was measured intraoperatively (ioPTH), and serum calcium was determined on the first postoperative day. A decrease of ≥50% of ioPTH and normalized serum calcium postoperatively were defined as surgical success. A total of 39 patients with pHPT were referred for FCH-PET/CT; 23 of these patients eventually had parathyroidectomy and a histopathological workup of the lesions and were included in the study (Figure 1).

### 18F-Fluorocholine-PET/CT

After intravenous injection of 18F-fluorocholine (median applied activity, 160 MBq; IQR, 180–149), with an uptake time of 45 min, images were obtained using a PET/CT (Discovery 600; GE Healthcare, USA). First, a contrast-enhanced CT was acquired from the base of the skull to the diaphragm. PET images were then obtained using a 3-min acquisition time per bed position. Images were sent to a dedicated workstation (AW, GE Healthcare) and the PET/CT images were analyzed by a physician who was board certified in radiology and nuclear medicine with 12 years of experience reading of PET/CT images. Focal lesions with significant tracer uptake on PET images and corresponding nodular lesions in CT, found in typical locations for orthotopic or heterotopic parathyroid tissues, were rated as positive PET/CT studies.

### Surgical Methods

23 included patients with pHPT underwent surgery in general anesthesia in our hospital. The surgical procedure was an open minimal invasive parathyroidectomy with recurrent laryngeal nerve monitoring in 21 patients. In two patients, ectopic/mediastinal adenoma resection was performed by a thoracoscopic approach. Depending on the diagnostic result of the preoperative FCH-PET/CT, unilateral or a bilateral neck exploration was done. In case of coexisting with a multinodular goiter, a thyroidectomy was also performed. The total intraoperative number of lesions found, and their location were recorded. To confirm the success of the surgical procedure resection, specimens were cut and frozen to determine the histological subtype: normal or hyperfunctioning parathyroid tissue. After introduction of general anesthesia and 7 min after surgical removal of the last enlarged tissue, PTH was determined to confirm its decrease. The procedure was defined successful if there was a decrease in the serum PTH of more than 50% from the baseline. Regular control of serum calcium and PTH was performed after surgery to document the success of the parathyroidectomy and to determine clinically relevant hypocalcemia symptoms. No operative complications in all patients were observed. The histopathological analysis served as the gold standard for comparison with the preoperative imaging results.

### Performance Analysis

FCH-PET/CT imaging results were compared with the intraoperative situs and the histopathological examination as the gold standard for the diagnosis of hyperfunctioning parathyroid tissue. The results of the FCH-PET/CT were classified as (a) true positive: the regional tracer uptake correlated with the histological results of hyperfunctioning parathyroid tissue, (b) false positive: a regional tracer uptake with histology other than hyperfunctioning parathyroid tissue, (c) false negative: an absent regional tracer uptake with a histology of hyperfunctioning parathyroid tissue, and (d) true negative: an absent regional tracer uptake and histological findings of normal parathyroid tissue.

### Statistical Analysis

Based on the performance analysis (cf. above), sensitivities were calculated on a per-patient and a per-lesion basis using the quotient of true positive/true positive + true negative. 95% confidence interval (CI) for sensitivity is calculated by the Clopper–Pearson method. If not stated otherwise, values are expressed as median and IQR (Q3–Q1).

## Results

The baseline characteristics of the patients are shown in Table 1. A total of 23 patients (18 females and 5 males) were referred for surgery, 4 of them needed repeated procedures. Four of the 10 patients with goiters had hemithyroidectomy, and 2 patients had total thyroidectomy. There was no uptake using ^99m^Tc-sestamibi scintigraphy/SPECT/CT in 19 patients and an undefined uptake in 4 patients. Six patients had equivocal lesions in the sonographic examination of the cervical region, and all other ultrasound examinations were negative. In 10 of 23 (43%) patients, multinodular goiters were present, and all patients had a normal thyroid function.

**Table 1:** Characteristics of the patients.

Parameter	Value^a^
Age (years)	61.9 (41–83)
Serum calcium preoperative (mmol/L)	2.69 (2.83–2.60)
PTH preoperative (pg/mL)	137 (220–104)
Serum calcium postoperative (mmol/L)	2.19 (2.32–2.07)
PTH postoperative (pg/mL)	35 (49–23)
ioPTH decrease (%)	79 (85–64)
Weight of adenoma/hyperplastic gland (g)^b^	0.95 (1.8–0.38)

*^a^All values are median values (IQR), except for age: mean (range)*.

*^b^Based on histopathological examinations*.

FCH-PET/CT showed 22 lesions, and 1 patient had negative imaging with FCH-PET/CT. A total of 29 parathyroid lesions were resected, and histopathological examinations revealed 24 instances of overfunctioning parathyroid tissues, with parathyroid glands with a normal histology. All patients were classified as a surgical success based on the decrease of ioPTH and normalization of postoperative serum calcium levels. The postoperative course was uneventful in all patients, with no complications from the surgery. The details of the imaging and the intraoperative and the histopathological findings are listed in Table 2.

**Table 2:** Laboratory findings, imaging, and histological results.

Subj. Nr.	Ca preop. (mmol/L)	Ca. postop. (mmol/L)	PTH intraop, before surgery (pg/mL)	PTH intraop, after surgery (pg/mL)	PTH first day after surgery (pg/mL)	Preop. US	Preop. sesta-MIBI	Preop. 18F-FCH	Nodular goiter	Resected lesions	Histology
1	2.76	2.13	120	20	48	–	–	P3R	–	P3R	P3R Ad
2	2.72	2.41	112	23	20	?P4R	–	P4R	–	P3R/P4R	P3R No/P4R HT
3	2.60	2.15	220	47	73	–	–	P4L	Yes	P3L/P4L	P3L No/P4L HT
4	2.60	2.36	89	36	42	–	–	P3R	Yes	P3R	P3R HT
5	2.75	2.17	127	33	26	–	?P4L	P4L	–	P4L	P4L HT
6	3.07	2.58	164	87	61	–	–	P3R	–	P3R	P3R HT
7	2.67	2.38	100	15	5	–	–	P3L	–	P3L/P4L	P3L No/P4L HT
8	2.76	2.37	423	125	19	–	–	P4R	–	P4R	P4R HT
9	2.65	2.25	128	22	50	–	–	P4R	Yes	P4R	P4R HT
10	2.65	2.27	148	28	49	?P3R	–	P3R	–	P3R	P3R HT
11	2.77	2.25	177	51	9	–	–	P4R	Yes	Hemitx R^a^	P4R HT
12	2.61	2.23	84	18	14	?P3R	–	P3R	Yes	P3R/P4R	P3R HT/P4R No
13	2.67	2.30	395	95	6	–	–	P3L	Yes	P3L	P3L HT
14	2.60	2.31	104	25	13	?P3R	–	–	–	P3R/P3L	P3R HT/P3L HT
15	2.75	2.16	119	17	13	–	–	Me	–	Me/Thy	Me/Thy HT
16	2.64	2.2	137	23	34	–	?P3R	P3R	–	P3R	P3R HT
17	2.51	2.2	68	33.8	47.8	–	–	P3L	Yes	P3L/P4L	P3L HT
18	2.5	2.18	157	72	42	–	–	P3R	Yes	P3R	P3R HT
19	3.03	2.45	473	104	21	–	–	Me	–	Me	Me HT
20	3.06	2.39	129	24	16	–	?P3L	P3L	Yes	P3L	P3L HT
21	2.55	2.12	100	12.2	16.5	–	–	P3L	–	P3L	P3L HT
22	2.73	1.66	203	30.5	17.7	?P3R	–	P3R	Yes	P3R	P3R HT
23	2.55	2.19	96	35	51	?P3L	?P3L	P3L	–	P3L	P3L HT

*^a^Intrathyroidal adenoma; ?, equivocal/doubtful; –, no localization possible/absent*.

*P3, inferior parathyroid gland; P4, superior parathyroid gland; R, right; L, left; No, normal parathyroid tissue; Hemitx, hemithyroidectomy; Me, mediastinal; HT, hyperfunctioning tissue; Thy, thymus*.

In 21 of 23 patients, hyperfunctioning parathyroid tissue (adenoma/hyperplasia) was correctly localized by FCH-PET/CT (21 true positive, 1 false negative, and 1 false positive). The patient (patient 14) with false-negative FCH-PET/CT had multiglandular disease. This translates into a per-patient sensitivity of 95.5% (95% CI, 77.2–99.9). On a per-lesion basis (29 lesions resected), there were 21 true positives, 3 false negatives, 1 false positive, and 4 true negatives, which accounted for a lesion-based sensitivity of 87.5% (95% CI, 67.6–97.3). Nine patients had equivocal/doubtful findings in the ultrasound and/or ^99m^Tc-sestamibi scintigraphy/SPECT/CT. In 8 of these 9 patients, FCH-PET/CT provided the correct diagnosis.

Despite the high prevalence of multinodular goiters, the diagnostic accuracy of the FCH-PET/CT was excellent, especially for thyroid nodules that did not lead to false-positive findings. In one case (patient 13), the thyroid gland showed diffusely enhanced uptake, but the adenoma was clearly circumscribed. In another case, there was focal uptake in the liver, but no other foci were found in males (i.e., prostate) or females (i.e., breast).

## Discussion

We report a retrospective cohort study of patients with pHPT and negative conventional imaging studies, who underwent 18F-fluorocholine-PET/CT as an additional imaging technique for localization.

**Figure 2:**
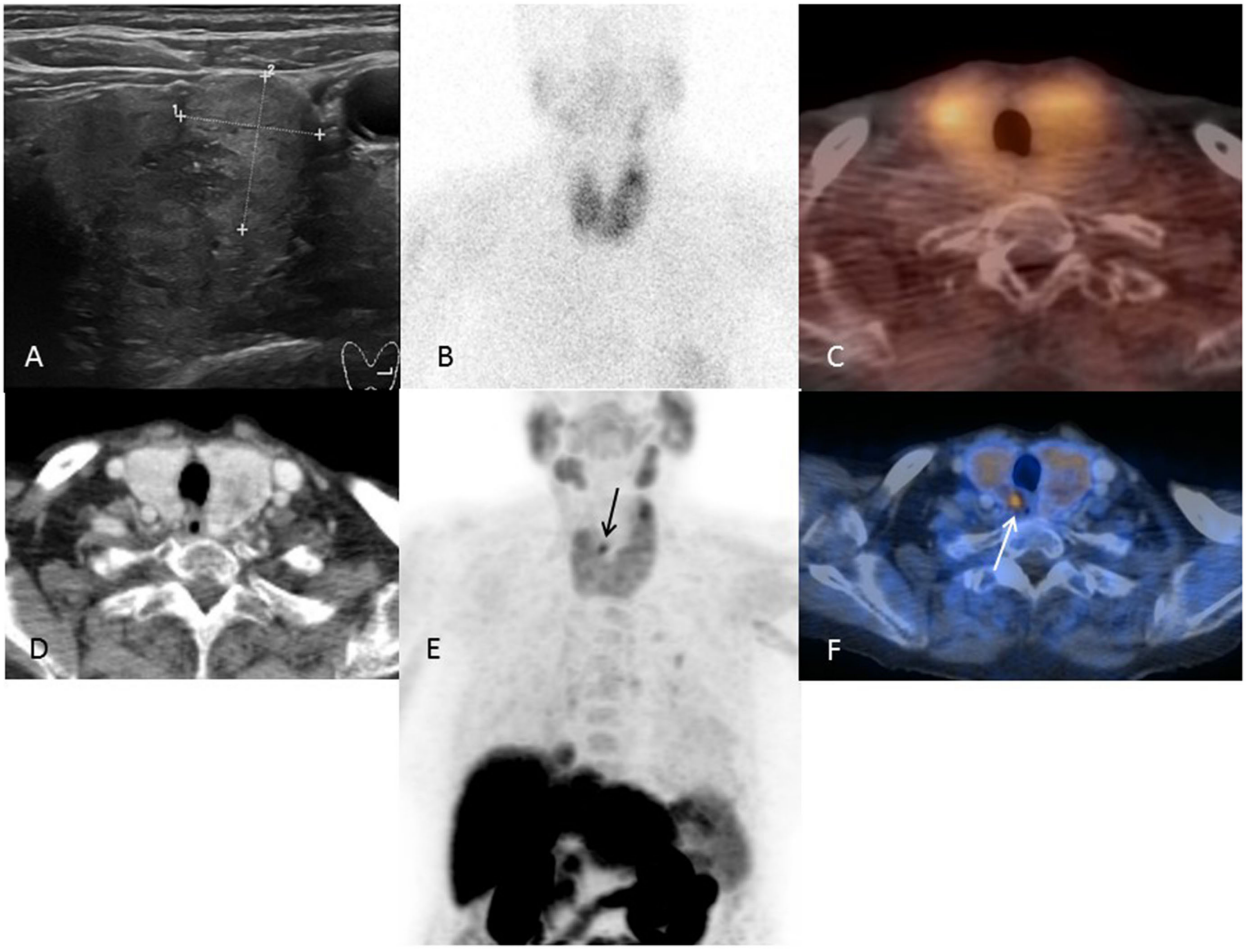
A representative case of a patient with multinodular goiter and negative conventional imaging. Images of an 82-year-old patient (patient number 9). Ultrasound **(A)** showing a bilateral multinodular goiter and no visible parathyroid adenoma. ^99m^Tc-sestamibi SPECT/CT **(B,C)** without detection of a parathyroid adenoma. FCH-PET/CT **(D–F)** with clear visualization of a small parathyroid retrotracheal adenoma at the upper right pole.

Our results showed the excellent ability of FCH-PET/CT to localize hyperfunctioning parathyroid tissue in patients with pHPT and negative or equivocal sestamibi/ultrasound findings (representative case: Figure 2). We confirmed the superior diagnostic ability of this newer functional imaging technique as reported in other studies (22, 23, 25). Compared to other studies (22, 23, 25), our study cohort was uniform, so the resulting localization accuracy may be representative of patients with sporadic pHPT and negative conventional imaging.

Our study cohort was characterized by a high prevalence of coexisting multinodular goiters or thyroid nodules (43%), which may be at least partially responsible for the high rate of negative imaging with ^99m^Tc-sestamibi scintigraphy/SPECT/CT and/or ultrasound. Previous studies have reported that the sensitivity of ^99m^Tc-sestamibi scintigraphy and/or ultrasound decreases in the presence of thyroid nodules or a multinodular goiter (26–29). However, in our patients with goiters, the FCH-PET/CT localized the lesions correctly within the “classical” positions.

The one patient with false-negative FCH-PET/CT followed by successful surgery had a multiglandular disease. Three excluded patients who had unsuccessful prior surgery were also negative using FCH-PET/CT (one patient had a known D418D polymorphism and two had MEN 1). This suggests that parathyroid multigland involvement in patients with pHPT may reduce the sensitivity and specificity of FCH-PET/CT, so further studies are needed using FCH-PET/CT for patients with pHPT because of a multigland disease, such as MEN 1/2 and D418D polymorphism.

Approximately (20–30%) of patients with pHPT and negative conventional imaging before parathyroidectomy are commonly found in daily clinical practice (30–32). When a focused parathyroidectomy is planned, some clinicians select additional diagnoses (31). Conventional MRI and CT have low detection rates and are generally not recommended (18). Four-dimensional CT provides an improved sensitivity of approximately 88% (33, 34), but results in a much higher radiation dose to the thyroid bed (35, 36). Furthermore, selective venous sampling is cumbersome and reserved for special situations (18).

Compared to scintigraphy and SPECT/CT, PET/CT offers better spatial resolution and better lesion to background ratios. In addition, PET can be easily combined with a diagnostic contrast-enhanced CT to provide the surgeon with important anatomical information regarding the localization of the parathyroid adenoma in relationship to other important structures such as the trachea, vessels, and the esophagus. With optimization of the PET/CT protocol, the radiation dose of FCH-PET/CT is comparable to SPECT/CT, using approximately 8 mSv (37).

The limitations of this study are threefold; it is a retrospective analysis, the sample size was small, and we had to exclude several patients from surgery. There was also a selection bias toward patients with negative/equivocal imaging.

In conclusion, our study showed that for patients with pHPT and negative/equivocal imaging with ^99m^Tc-sestamibi scintigraphy/ultrasound, FCH-PET/CT provides an excellent sensitivity of >90% per-patient and of >87% per lesion-based level. Furthermore, the diagnostic accuracy of FCH-PET/CT remains unchanged in the presence of thyroid nodules or multinodular goiters. However, larger cohorts are needed in future trials to confirm the findings of our study.

## Ethics Statement

The study conformed to the declaration of Helsinki and was approved by the local ethics committee (approval number: EKNZ-2017-00021).

## Author Contributions

Drafting of the study and the manuscript was carried out by all authors. Acquisition, analysis, and interpretation of the data were carried out by BN, WM, and JM (surgical part); KS and HG (imaging); and SF, IS-W, and CH (endocrinological studies and preoperative ultrasound). SF, IS-W, and CH were responsible for the writing of the manuscript. All authors gave the final approval of the version to be published.

## Conflict of Interest Statement

The authors declare that the research was conducted in the absence of any commercial or financial relationships that could be construed as a potential conflict of interest.
